# The Organochlorine *o,p’*-DDT Plays a Role in Coactivator-Mediated MAPK Crosstalk in MCF-7 Breast Cancer Cells

**DOI:** 10.1289/ehp.1104296

**Published:** 2012-05-18

**Authors:** Melyssa R. Bratton, Daniel E. Frigo, H. Chris Segar, Kenneth P. Nephew, John A. McLachlan, Thomas E. Wiese, Matthew E. Burow

**Affiliations:** 1 Department of Pharmacology, and; 2 Center for Bioenvironmental Research, Tulane University, New Orleans, Louisiana, USA; 3 Center for Nuclear Receptors and Cell Signaling, Department of Biology and Biochemistry, University of Houston, Houston, Texas, USA; 4 Department of Medicine, Section of Hematology and Medical Oncology, Tulane University Health Sciences Center, New Orleans, Lousiana, USA; 5 Department of Medical Sciences, Indiana University School of Medicine, Bloomington, Indiana, USA; 6 Division of Basic Pharmaceutical Sciences, College of Pharmacy, Xavier University of Louisiana, New Orleans, Louisiana, USA

**Keywords:** breast cancer, CBP, coactivator, crosstalk, DDT, dichlorodiphenyltrichloroethane, endocrine-disrupting chemical, HIF-1α, MAPK, organochlorine, p38 kinase, vascular endothelial growth factor

## Abstract

Background: The organochlorine dichlorodiphenyltrichloroethane (DDT), a known estrogen mimic and endocrine disruptor, has been linked to animal and human disorders. However, the detailed mechanism(s) by which DDT affects cellular physiology remains incompletely defined.

Objectives: We and others have shown that DDT activates cell-signaling cascades, culminating in the activation of estrogen receptor-dependent and -independent gene expression. Here, we identify a mechanism by which DDT alters cellular signaling and gene expression, independent of the estrogen receptor.

Methods: We performed quantitative polymerase chain reaction array analysis of gene expression in MCF-7 breast cancer cells using either estradiol (E_2_) or *o,p*´-DDT to identify distinct cellular gene expression responses. To elucidate the mechanisms by which DDT regulates cell signaling, we used molecular and pharmacological techniques.

Results: E_2_ and DDT treatment both altered the expression of many of the genes assayed, but up-regulation of vascular endothelial growth factor A (*VEGFA*) was observed only after DDT treatment, and this increase was not affected by the pure estrogen receptor α antagonist ICI 182780. Furthermore, DDT increased activation of the HIF-1 response element (HRE), a known enhancer of the *VEGFA* gene. This DDT-mediated increase in HRE activity was augmented by the coactivator CBP (CREB-binding protein) and was dependent on the p38 pathway.

Conclusions: DDT up-regulated the expression of several genes in MCF-7 breast cancer cells that were not altered by treatment with E_2_, including *VEGFA*. We propose that this DDT-initiated, ER-independent stimulation of gene expression is due to DDT’s ability to initiate crosstalk between MAPK (mitogen-activated protein kinase) signaling pathways and transcriptional coactivators.

Endocrine-disrupting chemicals (EDCs), such as polychlorinated biphenyls (PCBs), phthalates, phenolics, and other organochlorines, can affect the endocrine system by altering steroid receptor function, resulting in apparent estrogen-like activity and possible reproductive dysfunction (McLachlan 2001; McLachlan et al. 2006; Tilghman et al. 2010). The estrogen-like activity of the organochlorine pesticide dichlorodiphenyltrichloroethane (DDT) and its congeners was first shown > 50 years ago (Tullner 1961), yet the mechanism of action of DDT as a hormone remains an enigma (see McLachlan 2001 for review). Although its use has been restricted to use for mosquito control in developing countries with tropical climates, DDT remains active in the environment worldwide and bioaccumulates in the fat stores of animals and humans because of its lipophilic nature and chemical stability (Kelly et al. 2004). The DDT metabolite 1,1-dichloro-2,2-bis(*p*-chlorophenyl)ethylene (DDE) continues to be detected in human serum with a high frequency at concentrations up to and exceeding 1,000 μg/kg lipids (Cole et al. 2006). DDT and its metabolites have been associated with human diseases including type 2 diabetes (Codru et al. 2007; Rignell-Hydbom et al. 2007), testicular tumors (McGlynn et al. 2008), pancreatic cancer (Porta et al. 2008), endometrial cancer (Hardell et al. 2004), and breast cancer (Cocco et al. 2000; Rier and Foster 2002; Safe and Zacharewski 1997; Sasco 2003; Wolff et al. 1993), but mechanisms to explain these associations remain elusive.

DDT mimics the natural hormone estradiol (E_2_) and can bind to estrogen receptor α (ERα) (Ahlborg et al. 1995; Gulledge et al. 2001; Klotz et al. 1996; Kuiper et al. 1998). In addition, DDT exerts cellular effects independently of ERα. For example, we previously demonstrated that DDT and its active metabolites are capable of inducing AP-1 mediated transcription, in both ERα-positive and ERα-negative cells (Frigo et al. 2002). We have also shown that DDT activates transcription at multiple DNA response elements through p38-mediated phosphorylation and activation of the coactivators p300 (Bratton et al. 2009) and GRIP1 (Frigo et al. 2006). Using endometrial cells, we have shown that DDT can activate both the p38 and ERK1/2 (extracellular signal-regulated kinases 1/2) pathways, again independently of the ER (Frigo et al. 2004). Therefore, we hypothesized that treatment of MCF-7 breast cancer cells with DDT would result in an altered gene expression profile compared with cells treated with E_2_, and that this altered phenotype could provide clues regarding the molecular mechanism of DDT’s distinct effects on cell physiology.

## Materials and Methods

*Chemicals*. We purchased *o,p*´-DDT, *p,p*´-DDT, *o,p*´- and *p,p*´-dichlorodiphenyldichloroethane (DDD), *p,p*´-dichlorodiphenyl acetic acid (DDA), and *o,p*´- and *p,p*´-DDE from AccuStandard (New Haven, CT); 17β-estradiol (E_2_); all protease inhibitors; and porcine insulin from Sigma Chemical Company (St. Louis, MO); UO126 (an ERK inhibitor) from Promega (Madison, WI); SP600125 (a JNK inhibitor) from BIOMOL Research Laboratories Inc. (Plymouth Meeting, PA); and SB203580 (a p38α/β inhibitor) from EMD Biosciences (Billerica, MA). Dulbecco’s modified Eagle medium (DMEM), phenol-red free DMEM, fetal bovine serum (FBS), BME (basal medium Eagle) amino acids, MEM (minimum essential medium) amino acids, l-glutamine, penicillin, streptomycin, and sodium pyruvate were obtained from GibcoBRL (Gaitherburg, MD). We purchased charcoal-stripped FBS from HyClone (Logan, UT), Effectene from QIAGEN (Valencia, CA), and MPER (mammalian protein extraction reagent) from Pierce (Thermo Scientific, Rockford, IL).

*Plasmids*. Hypoxia-inducible factor 1 (HIF-1)-luciferase (HRE-luc) was donated by B.S. Beckman (Tulane University); CMV-GAL4 (negative control) was a gift from E. Flemington (Tulane University); and GAL4-CBP was donated by R. Goodman (Oregon Health Sciences University, Portland, OR). We purchased pFR-Luc [GAL4-luciferase (GAL4-luc) reporter] and pFC-MEK1 [CA-MKK1; constitutively active MAPK kinase (MKK) 1] from Stratagene (La Jolla, CA), and pcDNA3.1 from Invitrogen (Carlsbad, CA). pcDNA3-CA-MKK5 [CA-MKK5; constitutively active MAPK kinase (MKK) 5] and dominant-negative (DN) ERK2 (DN-ERK2) were gifts from J.-D. Lee (Scripps Research Institute, La Jolla, CA). pcDNA3-CA-MKK6 [CA-MKK6; constitutively active MAPK kinase (MKK) 6] and pcDNA3-CA-MKK7 [CA-MKK7; constitutively active MAPK kinase (MKK) 7] were gifts from J. Han (Scripps Research Institute). JNK1 and p38α MAPK DN mutants (DN-JNK1, DN-p38α) were provided by R. Davis (University of Massachusetts Medical School, Worcester, MA). GST (glutathione *S*-transferase) expression vector was purchased from Amersham Biosciences (Piscataway, NJ). pGEX-CBP1 (aa: 390-790) and pGEX-CBP3 (aa: 1990-2441) were gifts from R.G. Roeder (Rockefeller University, New York, NY). pGEX-CBP2 (aa:1680-1892) was generated by polymerase chain reaction (PCR) using HA-CBP (histone acetyltransferase–CREB-binding protein) full length (gift from R. Goodman, Oregon Health Sciences University) as a template. Resultant DNA was subcloned into the *Eco*R1/*Sal*1 site of pGEX-5X-1 (Amersham Pharmacia Biotech, Arlington Heights, IL).

*Cell culture.* ER-positive MCF-7 human breast carcinoma cells (Burow et al. 2000) and ER-negative human embryonic kidney (HEK) 293 cells (Kuiper et al. 1998) were maintained as previously described (Bratton et al. 2009; Rhodes et al. 2010). MCF-7 cells were grown for 48 hr in phenol red–free DMEM supplemented with 5% charcoal-stripped FBS and supplements but without insulin (5% charcoal-stripped DMEM), as previously described (Burow et al. 1999). Fulvestrant resistant MCF-7F cells were grown as previously described (Fan et al. 2006).

*Quantitative PCR (qPCR) array analysis.* MCF-7 cells were seeded in 6-well plates, and drug treatment was initiated after 24 hr. Cells were lysed 48 hr later, and total RNA was harvested using the RNeasy Mini Kit (QIAGEN). We used the RT^2^ First Strand cDNA kit (SABiosciences, Frederick, MD) to perform cDNA synthesis from total RNA according to the manufacturer’s protocol. qPCR was then performed on a BioRad IQ5 Real-Time PCR Detection System (Bio-Rad, Hercules, CA) using a 96-well RT^2^ Profiler PCR Array (Breast Cancer and Estrogen Receptor Signaling PCR Array; PAHS-005; QIAGEN). Generation and analysis of cycle threshold (Ct) values were performed according to manufacturer’s instructions for the array. Three independent arrays were run for each treatment; values are presented as fold change relative to several housekeeping genes (18S rRNA, *HPRT1*, *RPL13A*, *GAPDH*, and *ACTB*). qPCR of *VEGFA* mRNA was performed on samples of MCF-7 cells treated with either vehicle (i.e., DMSO), DDT, or DDT plus ICI 182780 (ICI) as previously described (Bratton et al. 2009). qPCR arrays of MCF-7F cells were run on samples isolated from three independent experiments using triplicate Breast Cancer and Estrogen Receptor Signaling PCR Arrays as previously described (Tilghman et al. 2012).

*Luciferase assays*. MCF-7 and HEK 293 cells were transfected as previously described (Bratton et al. 2010). A GAL4-luc reporter, along with an empty expression vector or a GAL4-CBP fusion, was transfected into HEK 293 cells. The cells were then treated with vehicle or different MAPK inhibitors for 1 hr, followed by addition of vehicle or 50 μM *o*,*p*´-DDT for 18 hr. Luciferase activity was measured in 100 μL of the lysed sample using a Berthold luminometer (Titertek Instruments Inc., Huntsville, AL) and 100 μL Bright Glo luciferase assay reagent (Promega, Madison, WI).

*GST-fusion protein purification and* in vitro *kinase assay*. The GST and GST-CBP fusion proteins were generated as previously described (Bratton et al. 2009). Roughly, 3–5 μg of eluted purified GST-fusion protein or 200 ng of purified mitogen-activated protein kinase (MAPK)-activated protein kinase-2 (Upstate Biotechnology, Lake Placid, NY) was phosphorylated by activated p38α as previously described (Bratton et al. 2009). Samples were analyzed by 4–12% SDS-PAGE (Invitrogen), stained with coomassie blue to monitor expression, and subjected to autoradiography as described by Bratton et al. (2010).

## Results

**Figure 1:**
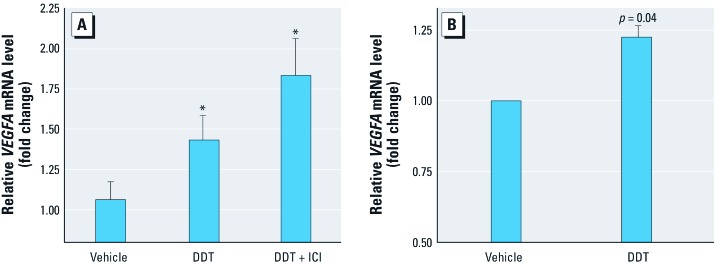
*VEGFA* expression in ERα-positive MCF‑7 cells incubated for 18 hr with vehicle, 10 μM DDT, or DDT + ICI (100 nM) (*A*) and ERα-negative MCF‑7F cells incubated for 18 hr with vehicle or 10 μM DDT (*B*). qPCR results are presented as fold change relative to housekeeping genes. *p < 0.05 compared with vehicle control (*n* = 3).

*DDT- and E_2_-induced gene expression*. We used a qPCR-based human breast cancer pathway array to compare gene expression in MCF-7 breast cancer cells after treatment with vehicle, 1 nM E_2_, or 10 μM *o,p*´-DDT for 18 hr. E_2_ and DDT both significantly altered the expression of 13 genes known to be involved in breast cancer signaling. Interestingly, several genes were differentially up-regulated by DDT compared with E_2_, including Fas ligand (*FASLG*), integrin alpha 6 (*ITGA6*), and vascular endothelial growth factor A [*VEGFA*; an important factor in cellular angiogenic control mechanisms and differentiation (Zhang et al. 1995)] [Table 1; see also Supplemental Material, Table S2 (http://dx.doi.org/10.1289/ehp.1104296)]. To address whether the effect of DDT on *VEGFA* expression in MCF-7 cells is dependent on E_2_ or ERα, we assayed *VEGFA* expression by qPCR in MCF-7 cells incubated in the presence of the ERα inhibitor ICI. Because ICI had no effect on the DDT-mediated increase in *VEGFA* expression in MCF-7 cells, we concluded that the effect of DDT was ERα independent (Figure 1A). Consistent with this hypothesis, we observed a statistically significant increase in *VEGFA* expression in ERα-negative MCF-7F cells in response to DDT (Figure 1B; see also Supplemental Material, Table S1).

**Table 1:** qPCR array analysis of MCF-7 cells.

Gene	Description	o,p’-DDT	p-Value (DDT/veh)	E2	p-Value (E2/veh)
Bcl-2		B-cell CLL/lymphoma 2		3.00		0.0011		2.65		0.0006
CCNA1		Cyclin A1		1.97		0.0444		1.94		0.0057
CTSD		Cathepsin D		2.96		0.0228		2.64		0.0431
FASLG		Fas ligand		2.61		0.0156		0.98		0.9500
FOSL1		FOS-like antigen 1		2.81		0.0002		2.72		0.0000
HMGB1		High-mobility group box 1		1.70		0.0172		1.44		0.0013
IL6R		Interleukin 6 receptor		2.11		0.0161		1.7		0.0548
ITGA6		Integrin, alpha 6		2.28		0.0376		1.47		0.1152
NGFR		Nerve growth factor receptor		1.49		0.0486		1.33		0.2321
NME1		Non-metastatic cells 1		2.46		0.0006		2.96		0.0000
PGR		Progesterone receptor		229		0.0000		152		0.0000
SCGB1D2		Secretoglobin, family 1D, member 2		6.88		0.0035		2.43		0.0511
SERPINA3		Serpin peptidase inhibitor, clade a, member 3		2.72		0.0139		2.62		0.0042
SERPINB5		Serpin peptidase inhibitor, clade b, member 5		4.70		0.0004		4.70		0.0004
SLC7A5		Solute carrier family 7, member 5		13.7		0.0002		11.62		0.0003
STC2		Stanniocalcin 2		5.46		0.0001		3.94		0.0000
TFF1		Trefoil factor 1		23.3		0.0000		28.93		0.0000
VEGFA		Vascular endothelial growth factor A		1.97		0.0474		1.63		0.1023
veh, vehicle. Significantly up‑regulated genes are shown with their corresponding p‑values (n = 3 separate arrays).										

**Figure 2:**
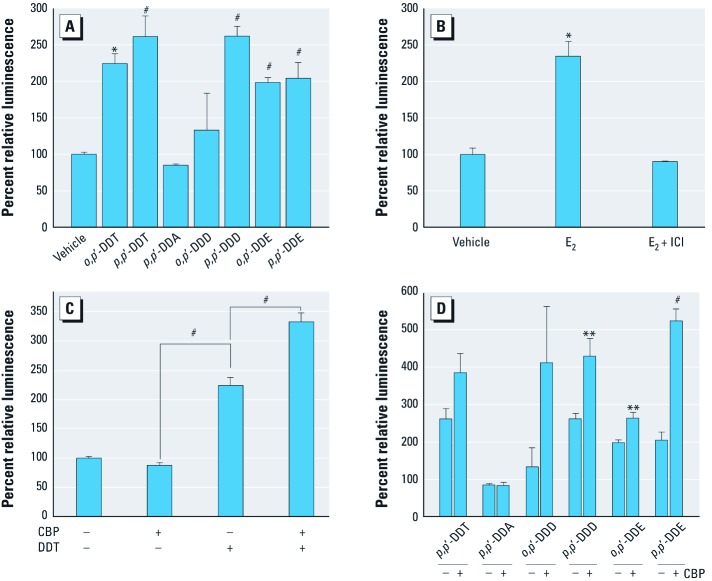
Organochlorines augment CBP activation of transcription from an HRE. (*A*) MCF‑7 cells transfected with an HRE-luc reporter and incubated with DDT metabolites (10 μM; *n* = 3). (*B*) MCF‑7 cells transfected with an HRE as in *A*, followed by incubation with vehicle, E_2_, or E_2_ + ICI (*n* = 3). (*C*) MCF‑7 cells transfected with an empty vector or a CBP expression vector plus an HRE-containing luciferase reporter and incubated overnight with vehicle or 10 μM *o,p’*-DDT (*n* = 3). (*D*) MCF‑7 cells treated as in *C* with metabolites 10 μM DDT (*n* = 4–6). Luminescence values are shown as the mean ± SE percentage of vehicle control, with the vehicle control set to 100%. *p < 0.05, **p < 0.01, and ^#^*p* < 0.001, compared with vehicle control (*A* and *B*), the metabolite without CBP (*D*), or as indicated (*C*).

*DDT and its metabolites activate the HIF-1 response element (HRE).* The *VEGFA* gene contains an estrogen responsive element (Kazi et al. 2005; Stoner et al. 2000, 2004) and is regulated by estrogens in mammary and uterine cells (Hyder et al. 1996; Nakamura et al. 1996, 1999). However, *VEGFA* expression is down-regulated by E_2_ in human breast cancer cells (Hyder et al. 1998). We previously showed that DDT stimulated transcription in ERα-negative human embryonic kidney cells by activating the HRE (Bratton et al. 2009). Because VEGFA contains an HRE within its promoter (Liu et al. 1995), we tested the effects of DDT and DDT metabolites on transcription of an HRE-luc reporter construct in MCF-7 breast cancer cells. Transcription was more than doubled in response to 10 μM *o,p*´-DDT (Figure 2A). HRE activity also increased significantly in response to the active metabolites *p,p*´-DDT, *p,p*´-DDD, *o,p*´-DDE, and *p,p*´-DDE, but not in response to the inactive metabolite *p,p*´-DDA (Figure 2A). E_2_ also activated the HRE-luc reporter in MCF-7 cells, but this effect was blocked by ICI (Figure 2B). This suggests that E_2_ can activate HREs; this is not surprising considering the general nature of the HRE reporter and the possibility that HREs are located within genes mediated by ERα–E_2_. Our cumulative results suggest that DDT alters *VEGFA* expression in MCF-7 cells in part by activating an HRE within the *VEGFA* promoter, in a manner independent of the ERα or E_2_. However, the fact that E_2_ stimulates an HRE reporter in MCF-7 cells leaves open the possibility that the DDT effect on *VEGFA* expression could be mediated, at least in part, through the ERα-E_2_ pathway.

*DDT potentiates CBP-induced transcriptional activation of the HRE.* CBP is a general transcriptional coactivator that functions to regulate gene expression through interaction with various transcription factors, including CREB (Giordano and Avantaggiati 1999), Elk 1 (Janknecht and Nordheim 1996), c-Jun (Giordano and Avantaggiati 1999), and TBP (TATA box binding protein) (Goodman and Smolik 2000). Based on previously published data showing a direct interaction between HIF-1 and CBP (Dames et al. 2002), we hypothesized that DDT activation of CBP may potentiate the activation of HRE-mediated transcription. HRE-luc activity was unchanged in MCF-7 cells transfected with CBP, but activity increased approximately 3.3 times following the addition of 10 μM DDT to CBP-transfected cells, compared with only a 2× increase in cells transfected with an empty vector (*p* < 0.001 for CBP-positive versus CBP-negative cells) (Figure 2C). Other DDT metabolites also enhanced activation of the HRE-luc construct in cells expressing CBP, with the exception of the negative metabolite control *p,p*-DDA (Figure 2D).

**Figure 3:**
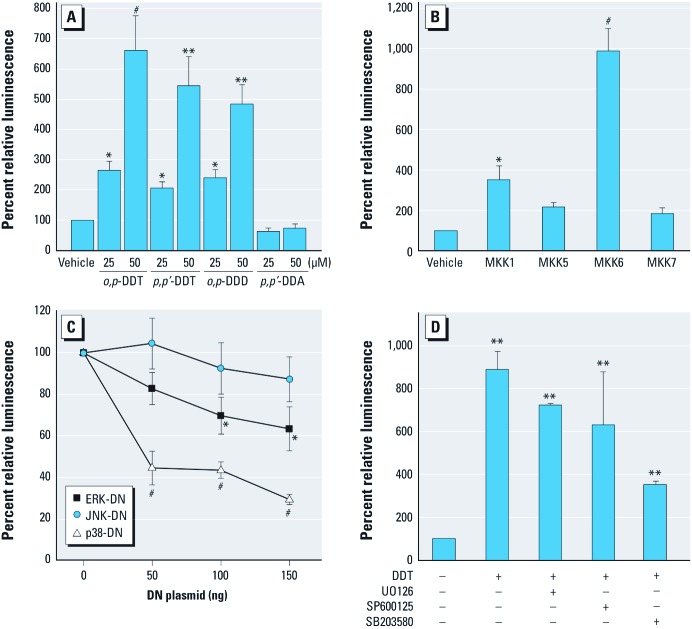
DDT and its metabolites stimulate the coactivator CBP through activation of the p38 MAPK pathway. (*A*) ER-negative HEK 293 cells transfected with GAL4-CBP and a GAL4-luc reporter and incubated overnight with organochlorines; *n* = 4. (*B*) HEK 293 cells transfected overnight with GAL4‑CBP, a GAL4-luc reporter, and either an empty vector or a vector expressing constitutively active MKK1 (ERK1/2), MKK5 (ERK5), MKK6 (p38), or MKK7 (JNK); *n* = 3. (*C*) HEK 293 cells transfected with GAL4-CBP, GAL4-luc, and increasing amounts of dominant negative (DN) mutants and then treated with 50 μM *o,p’*‑DDT for 24 hr; *n* = 4–6. (*D*) HEK 293 cells transfected with either empty vector or GAL4-CBP and GAL4-luc; after 6 hr, MAPK inhibitors were added (1 μM UO126, 1 μM SP600125, 6 μM SB203580), followed 1 hr later by 50 μM *o,p’*‑DDT for 18 hr (*n* = 4). Values are mean ± SE luciferase activity, with control values set to 100%. *p < 0.05, **p < 0.01, and ^#^*p* < 0.001, compared with vehicle control (*A*) or empty vector control (*B,C,D*).

*DDT and its active metabolites potentiate CBP activity.* HIF-1 forms a complex with CBP that increases CBP’s transactivation potential (Arany et al. 1996; Dames et al. 2002; Ema et al. 1999). We tested effects of DDT on CBP activity using a mammalian one-hybrid assay in which the full-length CBP is tethered to GAL4-DBD in conjunction with a GAL4 responsive luciferase reporter. Because our results suggested that the effect of DDT on *VEGFA* expression was ERα-independent, we used ERα-negative HEK 293 cells for this and subsequent experiments. The active DDT metabolites *o,p*´-DDT, *p,p*´-DDT, and *o,p*´-DDD potentiated CBP transactivation in a dose-dependent manner, whereas the inactive DDT metabolite, *p,p*´-DDA, had no effect (Figure 3A).

*DDT activation of CBP is dependent on the p38*α *MAPK pathway.* We previously demonstrated that AP-1 stimulation by DDT is dependent upon the p38α MAPK cascade (Frigo et al. 2004). Therefore, we tested the role of individual MAPK signaling pathways on DDT’s activation of CBP. HEK 293 cells were transfected with GAL4-CBP and either empty vector or vectors overexpressing constitutively active MKK1, MKK5, MKK6, or MKK7 mutants that selectively activate ERK1/2, ERK5, p38α, and JNK (respectively). MKK6, and to a lesser extent MKK1, potentiated CBP activity (Figure 3B). We next tested whether p38α was necessary for DDT-induced activation of CBP in HEK 293 cells transfected with GAL4-CBP (a GAL4-luc reporter) and increasing concentrations of DN-p38α, DN-ERK1/2, or DN-JNK1 in the presence of 50 μM *o,p*´-DDT. DDT-mediated activation of CBP was significantly inhibited in the absence of p38α-DN expression and to a lesser extent by ERK1/2-DN (Figure 3C). To confirm our molecular findings, we blocked DDT-induced coactivator activity with pharmacological inhibitors of the MAPK pathways. A GAL4-luc reporter, along with an empty expression vector or a GAL4-CBP fusion, was transfected into HEK 293 cells. The cells were then treated with vehicle or different MAPK inhibitors for 1 hr, followed by addition of vehicle or 50 μM *o,p*´-DDT for 18 hr. The p38α/β inhibitor SB203580 significantly blocked (*p* < 0.01) *o,p*´-DDT induction of CBP activity (Figure 3D), whereas neither the ERK inhibitor UO126 nor the JNK inhibitor SP600125 had a significant effect (Figure 3D). Collectively, these data confirm that DDT activates the transcriptional coactivator CBP via the p38 MAPK pathway.

**Figure 4:**
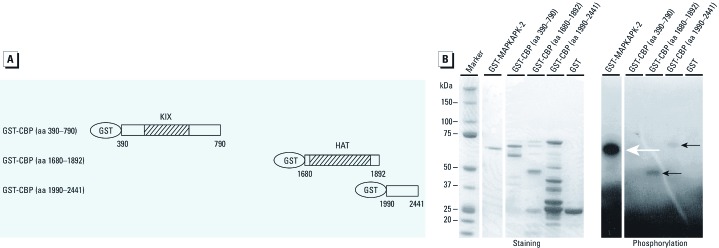
Activated p38α phosphorylates CBP *in vitro*. (*A*) Schematic of GST fusion proteins used for the *in vitro* kinase assays; aa, amino acids. (*B*) GST fusion proteins were purified and standardized according to protein concentration. Purified activated *p*38α was used to phosphorylate GST-CBP fragments in the presence of ^32^P-ATP followed by SDS‑PAGE and coomassie staining (left). Gels were dried and autoradiographed (right). GST-MAPKAPK2 was used as a positive control for *p*38α phosphorylation. The white arrow indicates phosphorylated GST-MAPKAPK2, and black arrows indicate phosphorylated GST-CBP fragments. Similar results were obtained in three independent experiments.

**Figure 5:**
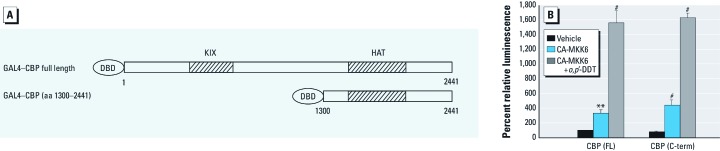
DDT and p38α target the C‑terminal of CBP. (*A*) Schematic of GAL4-CBP fusion constructs used for mammalian one-hybrid analysis; aa, amino acids. (*B*) HEK 293 cells transfected for 6 hr with full-length GAL4-CBP (FL) or the C‑terminal (C‑term) fragment GAL4-CBP (aa 1300-2441) plus GAL4‑luc. Some cells were also transfected with the CA-MKK6 mutant; other cells were transfected with MKK6-CA and incubated overnight with 50 μM *o,p’*‑DDT. Values represent the percent change (mean ± SE; *n* = 4) in CBP activity, with the vehicle set to 100%. **p < 0.01, and ^#^*p* < 0.001 compared with vehicle control.

*DDT induces the p38*α*-mediated phosphorylation and transcriptional activation of CBP*. Various kinases have been shown to potentiate CBP by phosphorylation (Ait-Si-Ali et al. 1999; Constantinescu et al. 2004). We hypothesized that p38α MAPK directly phosphorylates CBP, leading to its potentiation. To test this, we bacterially expressed recombinant CBP fused to GST for purification (Figure 4A) and subjected the purified proteins to an *in vitro* kinase assay in the presence of ^32^P (phosphorus-32) and activated p38α MAPK. The C-terminal fragments of CBP containing amino acids 1680–1892 and, to a lesser extent, 1990–2441 were phosphorylated by activated p38α, whereas the N-terminal fragment (amino acids 390–790) was not (Figure 4B). Activation of the C-terminal of CBP by DDT was tested using a deletion mutant of CBP containing amino acids 1300–2441 in a GAL4 fusion vector (Figure 5A). We overexpressed either the empty vector or a constitutively active MKK6 mutant in HEK 293 cells, in the presence or absence of *o,p*´-DDT. MKK6 activated the C-terminal of CBP in the absence of *o,p*´-DDT, but CBP activity was further augmented with the addition of 50 μM *o,p*´-DDT (Figure 5B). Taken together, these results suggest that DDT augments p38 activity, which in turn phosphorylates CBP within its C-terminal, resulting in increased CBP transcriptional activation.

## Discussion

Although estrogenic activity of DDT has been reported (Ahlborg et al. 1995; Gulledge et al. 2001; Klotz et al. 1996; Kuiper et al. 1998), the mechanism underlying the hormone activity of the organochlorine pesticide remains unclear. We have previously shown that DDT and its metabolites activate transcription factors such as AP-1 independently of ERα (Bratton et al. 2009; Frigo et al. 2004). In the present study, we further investigated the molecular differences in hormone action between DDT and E_2_ and characterized the qualities of DDT compared with other compounds that display estrogen-like properties. Although DDT and E_2_ both stimulated the transcription of a subset of ERα-regulated genes, including *Bcl-2, PgR,* and trefoil factor 1 (*TFF1*), DDT also up-regulated genes that were not affected by E_2_, including *FASLG*, *ITGA6*, and *VEGFA* (Table 1).

Differential gene expression induced by “estrogenic” environmental contaminants has been reported. For example, Goodson and colleagues treated nonmalignant high-risk donor breast epithelial cells (HRBECs) with E_2_ and BPA; using global gene expression analysis, they determined that BPA produced a distinct gene expression pattern compared with E_2_ (Dairkee et al. 2008; Goodson et al. 2011). Han et al. (2010) recently reported that DDT up-regulated aromatase gene expression in MCF-7 cells independently of ER function. Results of the present study also suggest that DDT is capable of altering gene expression in breast cancer cells in a manner different from that of E_2_.

Our gene expression analysis revealed that DDT up-regulated *VEGFA*, an important factor in angiogenic cell response and regulation, as well as cell differentiation (Zhang et al. 1995). DDT increased *VEGFA* expression in MCF-7 cells, even in the presence of the pure antiestrogen ICI, suggesting that the DDT effect is ERα independent. In addition, DDT increased *VEGFA* expression in the ERα-negative MCF-7F cell line. Although crosstalk can occur between DDT signaling estrogen response elements, as previously shown (Bratton et al. 2009), the results presented here strongly suggest that DDT-altered *VEGFA* expression in MCF-7 breast cancer cells is ERα independent.

Our results also suggest that DDT and its metabolites potentiate the activity of HIF-1α, which is known to bind the *VEGFA* promoter (Liu et al. 1995). However, because E_2_ activated the HRE reporter, ERα-independent effects of E_2_ on HRE activation and *VEGFA* expression remain a possibility. We have previously shown that DDT can regulate gene expression through the phosphorylation of coregulatory proteins such as SRC-2/GRIP1 (glucocorticoid receptor-interacting protein 1, steroid receptor coactivator-2), a member of the NCoA family of coregualtors (Frigo et al. 2006). Here, we demonstrated that active DDT compounds increased CBP activity and CBP-mediated transactivation of an HRE-linked reporter gene. DDT concentrations used in our experiments (10–50 μM) may appear high, but DDT metabolite levels > 20 ng/mL in blood (equivalent to 63 μM) have been reported (Longnecker et al. 2002; López-Carrillo et al. 2001; Martin et al. 2002), as well as levels > 4 mM in soils throughout North America (Aigner et al. 1998; Falconer et al. 1997; U.S. Geological Survey 2001). These results, taken together, support a role for DDT in activation of the CBP–HIF-1 complex and suggest a mechanism by which DDT increases *VEGFA* expression.

**Figure 6:**
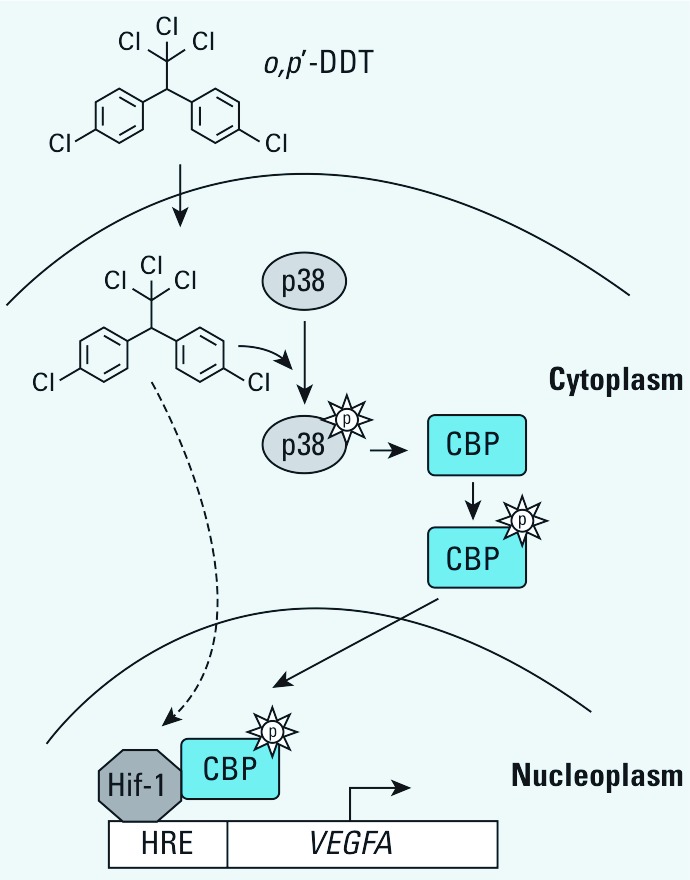
Proposed mechanism of organochlorine-mediated up‑regulation of *VEGFA* expression. We propose a mechanism whereby *o,p’*-DDT stimulates phosphorylation of p38 kinase, which in turn phosphorylates CBP. The activated CBP binds HIF-1, and this complex binds to the *VEGFA* promoter at the HRE, thereby increasing transcription of *VEGFA*.

We used both molecular and pharmacological tools to investigate the role of MAPK pathways in the DDT–CBP–HIF-1 signaling cascade. We showed that activation of the p38 pathway potentited CBP activity and that DDT’s effect on CBP activation was inhibited by blocking p38α. Finally, we showed that p38α directly phosphorylated the C-terminal of CBP,and that p38 activated CBP via its C-terminal region. These data, in conjunction with published reports of a direct interaction between the coactivator CBP and HIF-1α (Dames et al. 2002) suggest a mechanism for the expression of *VEGFA* in MCF-7 cells following DDT exposure: DDT activates p38, which leads to phosphorylation of CBP and enhanced binding to HIF-1α; the resulting HIF-1α–CBP complex binds to *VEGFA* promoter, increasing its transcription (Figure 6).

## Conclusions

Overall, our data demonstrate a link between organochlorine-mediated cell signaling through a MAPK pathway and the direct phosphorylation and regulation of coactivator function. These data suggest that coactivator phosphorylation might serve as a cellular sensor of environmental stress and lead to the modulation of key sets of adaptive genes. Moreover, these results suggest a possible mechanism by which environmental compounds may exert more, or less, E_2_-like potency than their ERα affinity implies.

## Supplemental Material

(295 KB) PDF
