# Supplementary material for: The Organochlorine o,p’-DDT Plays a Role in Coactivator-Mediated MAPK Crosstalk in MCF-7 Breast Cancer Cells
Source: Environ Health Perspect. 2012 May 18;120(9):1291–6. doi: 10.1289/ehp.1104296 (PMC3440107; doi:10.1289/ehp.1104296)
Supplement: (295 KB) PDF [file ehp.1104296.s001.pdf]

Supplemental Material

The Organochlorine o,p'-DDT Plays a Role in Coactivator-Mediated MAPK Crosstalk in MCF-7 Breast Cancer Cells

Melyssa R. Bratton, Daniel E. Frigo, H. Chris Segar, Kenneth P. Nephew, John A. McLachlan, Thomas E. Wiese, Matthew E. Burow

Supplemental Material, Table S1. qPCR array of MCF-7F cells.

Gene symbol	Description	Fold Regulation (DDT/Veh)	p value
AR	Androgen receptor	-9.0317	0.440419
BAD	BCL2-associated agonist of cell death	-15.366	0.208434
BAG1	BCL2-associated athanogene	-15.7251	0.253679
BCL2	B-cell CLL/lymphoma 2	-1.3582	0.03829
BCL2L2	BCL2-like 2	1.1961	0.052241
C3	Complement component 3	-1.1554	0.782079
CCNA1	Cyclin A1	1.0413	0.542735
CCNA2	Cyclin A2	-1.4557	0.011769
CCND1	Cyclin D1	-1.0058	0.972787
CCNE1	Cyclin E1	-1.21	0.23968
CD44	CD44 molecule (Indian blood group)	1.5422	0.002891
CDH1	Cadherin 1, type 1, E-cadherin (epithelial)	-1.21	0.205846
CDKN1A	Cyclin-dependent kinase inhibitor 1A (p21, Cip1)	-2.5344	0.90007
CDKN1B	Cyclin-dependent kinase inhibitor 1B (p27, Kip1)	-1.4557	0.00237
CDKN2A	Cyclin-dependent kinase inhibitor 2A (melanoma, p16, inhibits CDK4)	-1.5245	0.109123
CLDN7	Claudin 7	-1.5966	0.002555
CLU	Clusterin	-2.9113	0.021252
COL6A1	Collagen, type VI, alpha 1	1.0175	0.806653
CTNNB1	Catenin (cadherin-associated protein), beta 1, 88kDa	-1.5601	0.004348
CTSB	Cathepsin B	-1.078	0.437803
CTSD	Cathepsin D	-1.21	0.063807
CYP19A1	Cytochrome P450, family 19, subfamily A, polypeptide 1	1.2241	0.862951
DLC1	Deleted in liver cancer 1	-2.8448	0.010782
EGFR	Epidermal growth factor receptor	-1.5245	0.004899
ERBB2	V-erb-b2 erythroblastic leukemia viral oncogene homolog 2, neuro/glioblastoma derived oncogene homolog (avian)	-9.9062	0.070662
ESR1	Estrogen receptor 1	-9.0317	0.122889
ESR2	Estrogen receptor 2 (ER beta)	-1.2672	0.401208
FAS	Fas (TNF receptor superfamily, member 6)	-1.129	0.09034
FASLG	Fas ligand (TNF superfamily, member 6)	-1.6339	0.066675
FGF1	Fibroblast growth factor 1 (acidic)	1.4061	0.291349
FLRT1	Fibronectin leucine rich transmembrane protein 1	-1.0058	0.986079
FOSL1	FOS-like antigen 1	1.4726	0.009182

Supplemental Material, Table S1 (cont.)

GABRP	Gamma-aminobutyric acid (GABA) A receptor, pi	-3.1932	0.003233
GATA3	GATA binding protein 3	-1.4557	0.00488
GNAS	GNAS complex locus	-3.1932	0.002396
GSN	Gelsolin	-1.078	0.416832
HMGB1	High mobility group box 1	2.7479	0.7239
HSPB1	Heat shock 27kDa protein 1	-68.9909	0.416565
ID2	Inhibitor of DNA binding 2, dominant negative helix-loop-helix protein	-1.3272	0.077236
IGFBP2	Insulin-like growth factor binding protein 2, 36kDa	-1.7921	0.016659
IL2RA	Interleukin 2 receptor, alpha	1.116	0.50141
IL6	Interleukin 6 (interferon, beta 2)	1.374	0.053573
IL6R	Interleukin 6 receptor	1.4726	0.041435
IL6ST	Interleukin 6 signal transducer (gp130, oncostatin M receptor)	-1.129	0.097104
ITGA6	Integrin, alpha 6	1.4061	0.000454
ITGB4	Integrin, beta 4	-1.834	0.004426
JUN	Jun proto-oncogene	-1.4557	0.056947
KIT	V-kit Hardy-Zuckerman 4 feline sarcoma viral oncogene homolog	-1.6339	0.066675
KLF5	Kruppel-like factor 5 (intestinal)	1.0413	0.840779
KLK5	Kallikrein-related peptidase 5	-1.6339	0.002613
KRT18	Keratin 18	-1.6339	0.000497
KRT19	Keratin 19	-1.1554	0.082243
MAP2K7	Mitogen-activated protein kinase kinase 7	-1.1824	0.380596
MKI67	Antigen identified by monoclonal antibody Ki-67	-39.6248	0.06399
MT3	Metallothionein 3	-1.6339	0.066675
MUC1	Mucin 1, cell surface associated	-1.4897	0.014666
NFYB	Nuclear transcription factor Y, beta	-1.3272	0.000844
NGF	Nerve growth factor (beta polypeptide)	-1.6339	0.066675
NGFR	Nerve growth factor receptor	-2.7798	0.000196
NME1	Non-metastatic cells 1, protein (NM23A) expressed in	1.0905	0.235201
PAPPA	Pregnancy-associated plasma protein A, pappalysin 1	-1.6339	0.066675
PGR	Progesterone receptor	-1.6339	0.066675
PLAU	Plasminogen activator, urokinase	-1.129	0.425072
PTEN	Phosphatase and tensin homolog	-1.0534	0.264509

Supplemental Material, Table S1 (cont.)

PTGS2	Prostaglandin-endoperoxide synthase 2 (prostaglandin G/H synthase and cyclooxygenase)	-1.6339	0.066675
RAC2	Ras-related C3 botulinum toxin substrate 2 (rho family, small GTP binding protein Rac2)	-1.6339	0.066675
RPL27	Ribosomal protein L27	1.2527	0.067928
SCGB1D2	Secretoglobin, family 1D, member 2	-1.078	0.618955
SCGB2A1	Secretoglobin, family 2A, member 1	1.6915	0.113076
SERPINA3	Serpin peptidase inhibitor, clade A (alpha-1 antiproteinase, antitrypsin), member 3	1.3119	0.061166
SERPINB5	Serpin peptidase inhibitor, clade B (ovalbumin), member 5	1.2241	0.157399
SERPINE1	Serpin peptidase inhibitor, clade E (nexin, plasminogen activator inhibitor type 1), member 1	1.2819	0.040936
SLC7A5	Solute carrier family 7 (amino acid transporter light chain, L system), member 5	1.2819	0.191025
SPRR1B	Small proline-rich protein 1B	-4.9531	0.730958
STC2	Stanniocalcin 2	1.8129	0.001576
TFF1	Trefoil factor 1	1.4389	0.010301
TGFA	Transforming growth factor, alpha	-1.1554	0.340467
THBS1	Thrombospondin 1	-1.1554	0.122357
THBS2	Thrombospondin 2	-1.3272	0.025363
TIE1	Tyrosine kinase with immunoglobulin-like and EGF-like domains 1	-1.5245	0.089397
TNFAIP2	Tumor necrosis factor, alpha-induced protein 2	-1.5966	0.018487
TOP2A	Topoisomerase (DNA) II alpha 170kDa	-1.4224	0.00628
TP53	Tumor protein p53	-1.3899	0.058272
VEGFA	Vascular endothelial growth factor A	1.2241	0.041647
B2M	Beta-2-microglobulin	127.2628	0.667939

Supplemental Material, Table S1. qPCR array analysis of MCF-7F cells. qPCR arrays of MCF-7F cells treated with either vehicle or 10µM DDT were run on samples isolated from three independent experiments using triplicate Breast Cancer & Estrogen Signaling PCR Arrays.

Supplemental Material, Table S2. qPCR array of MCF-7 cells.

Gene symbol	Description	Fold Regulation (DDT/Veh)	p value	Fold Regulation (E_2/Veh)	p value
AR	Androgen receptor	0.73	0.0285	0.70	0.0261
BAD	BCL2-associated agonist of cell death	1.06	0.8421	0.79	0.4956
BAG1	BCL2-associated athanogene	0.59	0.1056	0.52	0.0207
BCL2	B-cell CLL/lymphoma 2	3.00	0.0011	2.65	0.0006
BCL2L2	BCL2-like 2	1.22	0.4896	1.10	0.7312
C3	Complement component 3	0.81	0.6753	0.55	0.1100
CCNA1	Cyclin A1	1.97	0.0444	1.94	0.0057
CCNA2	Cyclin A2	1.28	0.1708	1.41	0.0085
CCND1	Cyclin D1	2.28	0.0656	1.16	0.7079
CCNE1	Cyclin E1	0.81	0.2212	0.81	0.2008
CD44	CD44 molecule (Indian blood group)	1.57	0.0736	1.55	0.0529
CDH1	Cadherin 1, type 1, E-cadherin (epithelial)	0.76	0.0010	0.78	0.0179
CDKN1A	Cyclin-dependent kinase inhibitor 1A (p21, Cip1)	0.69	0.2083	0.91	0.6773
CDKN1B	Cyclin-dependent kinase inhibitor 1B (p27, Kip1)	1.01	0.9595	0.88	0.6278
CDKN2A	Cyclin-dependent kinase inhibitor 2A (melanoma, p16, inhibits CDK4)	1.51	0.6186	0.82	0.4367
CLDN7	Claudin 7	1.23	0.4211	0.97	0.9236
CLU	Clusterin	0.21	0.0001	0.11	0.0000
COL6A1	Collagen, type VI, alpha 1	0.68	0.0833	0.46	0.0008
CTNNB1	Catenin (cadherin-associated protein), beta 1, 88kDa	0.80	0.3459	0.70	0.1403
CTSB	Cathepsin B	0.63	0.1371	0.60	0.0041
CTSD	Cathepsin D	2.96	0.0228	2.64	0.0431
CYP19A1	Cytochrome P450, family 19, subfamily A, polypeptide 1	1.59	0.3552	1.15	0.7322
DLC1	Deleted in liver cancer 1	0.85	0.4211	0.62	0.0170
EGFR	Epidermal growth factor receptor	0.46	0.0146	0.45	0.0060
ERBB2	V-erb-b2 erythroblastic leukemia viral oncogene homolog 2, neuro/glioblastoma derived oncogene homolog (avian)	0.46	0.0294	0.33	0.0061
ESR1	Estrogen receptor 1	0.71	0.2677	0.49	0.0406
ESR2	Estrogen receptor 2 (ER beta)	0.87	0.4867	0.68	0.0994
FAS	Fas (TNF receptor superfamily, member 6)	1.35	0.1461	1.15	0.4104
FASLG	Fas ligand (TNF superfamily, member 6)	2.61	0.0156	0.98	0.9500
FGF1	Fibroblast growth factor 1 (acidic)	0.64	0.0916	0.40	0.0032
FLRT1	Fibronectin leucine rich transmembrane protein 1	1.38	0.5102	0.94	0.8911
FOSL1	FOS-like antigen 1	2.81	0.0002	2.72	0.0000
GABRP	Gamma-aminobutyric acid (GABA) A receptor, pi	0.16	0.0152	0.10	0.0001

Supplemental Material, Table S2 (cont.)

GATA3	GATA binding protein 3	1.01	0.9697	0.79	0.4808
GNAS	GNAS complex locus	0.61	0.1166	0.56	0.0360
GSN	Gelsolin	0.36	0.0008	0.38	0.0003
HMGB1	High mobility group box 1	1.70	0.0172	1.44	0.0013
HSPB1	Heat shock 27kDa protein 1	0.87	0.4067	0.99	0.9174
ID2	Inhibitor of DNA binding 2, dominant negative helix-loop-helix protein	0.87	0.5347	0.79	0.1426
IGFBP2	Insulin-like growth factor binding protein 2, 36kDa	0.80	0.6142	0.78	0.5921
IL2RA	Interleukin 2 receptor, alpha	0.79	0.4674	1.01	0.9635
IL6	Interleukin 6 (interferon, beta 2)	0.54	0.0456	0.72	0.1373
IL6R	Interleukin 6 receptor	2.11	0.0161	1.70	0.0548
IL6ST	Interleukin 6 signal transducer (gp130, oncostatin M receptor)	1.06	0.7558	0.83	0.3089
ITGA6	Integrin, alpha 6	2.28	0.0376	1.47	0.1152
ITGB4	Integrin, beta 4	0.52	0.0346	0.45	0.0138
JUN	Jun proto-oncogene	1.23	0.6386	1.02	0.9488
KIT	V-kit Hardy-Zuckerman 4 feline sarcoma viral oncogene homolog	0.73	0.4273	0.82	0.4367
KLF5	Kruppel-like factor 5 (intestinal)	0.58	0.0120	0.46	0.0012
KLK5	Kallikrein-related peptidase 5	0.60	0.4859	0.83	0.6677
KRT18	Keratin 18	0.44	0.0176	0.42	0.0027
KRT19	Keratin 19	0.98	0.8705	1.19	0.2614
MAP2K7	Mitogen-activated protein kinase kinase 7	1.43	0.3934	0.78	0.5801
MKI67	Antigen identified by monoclonal antibody Ki-67	1.09	0.7367	1.07	0.7722
MT3	Metallothionein 3	1.16	0.7076	0.91	0.7190
MUC1	Mucin 1, cell surface associated	0.58	0.0901	0.44	0.0202
NFYB	Nuclear transcription factor Y, beta	0.77	0.2585	0.69	0.0027
NGF	Nerve growth factor (beta polypeptide)	0.63	0.1830	0.82	0.4367
NGFR	Nerve growth factor receptor	1.49	0.0486	1.33	0.2321
NME1	Non-metastatic cells 1, protein (NM23A) expressed in	2.46	0.0006	2.96	0.0000
PAPPA	Pregnancy-associated plasma protein A, pappalysin 1	0.56	0.0647	0.72	0.1744
PGR	Progesterone receptor	229.01	0.0000	152.01	0.0000
PLAU	Plasminogen activator, urokinase	0.32	0.0017	0.29	0.0003
PTEN	Phosphatase and tensin homolog	0.86	0.2443	0.86	0.1711
PTGS2	Prostaglandin-endoperoxide synthase 2 (prostaglandin G/H synthase and cyclooxygenase)	2.38	0.4210	0.82	0.4367

RAC2	Ras-related C3 botulinum toxin substrate 2 (rho family, small GTP binding protein Rac2)	3.21	0.1364	1.55	0.1196
RPL27	Ribosomal protein L27	1.06	0.8228	1.22	0.3457
SCGB1D2	Secretoglobin, family 1D, member 2	6.88	0.0035	2.43	0.0511
SCGB2A1	Secretoglobin, family 2A, member 1	1.36	0.3466	1.05	0.8463
SERPINA3	Serpin peptidase inhibitor, clade A (alpha-1 antiproteinase, antitrypsin), member 3	2.72	0.0139	2.62	0.0042
SERPINB5	Serpin peptidase inhibitor, clade B (ovalbumin), member 5	4.70	0.0004	4.70	0.0004
SERPINE1	Serpin peptidase inhibitor, clade E (nexin, plasminogen activator inhibitor type 1), member 1	0.35	0.0181	0.42	0.0139
SLC7A5	Solute carrier family 7 (amino acid transporter light chain, L system), member 5	13.72	0.0002	11.62	0.0003
SPRR1B	Small proline-rich protein 1B	1.24	0.6745	1.75	0.0664
STC2	Stanniocalcin 2	5.46	0.0001	3.94	0.0000
TFF1	Trefoil factor 1	23.30	0.0000	28.93	0.0000
TGFA	Transforming growth factor, alpha	1.09	0.6578	0.94	0.7549
THBS1	Thrombospondin 1	0.62	0.0277	0.80	0.1801
THBS2	Thrombospondin 2	0.78	0.2588	0.61	0.0165
TIE1	Tyrosine kinase with immunoglobulin-like and EGF-like domains 1	0.67	0.2734	0.82	0.4367
TNFAIP2	Tumor necrosis factor, alpha-induced protein 2	0.26	0.0021	0.23	0.0007
TOP2A	Topoisomerase (DNA) II alpha 170kDa	0.97	0.9093	0.91	0.6314
TP53	Tumor protein p53	0.90	0.5926	0.88	0.5090
VEGFA	Vascular endothelial growth factor A	1.97	0.0474	1.63	0.1023
B2M	Beta-2-microglobulin	1.02	0.8612	0.79	0.0374

Supplemental Material, Table S2. qPCR array analysis of MCF-7 cells. qPCR arrays of MCF-7 cells treated with either vehicle,10μM DDT, or 1 nM E_2 were run on samples isolated from three independent experiments using triplicate Breast Cancer & Estrogen Signaling PCR Arrays.
